# Sam68 Mediates the Activation of Insulin and Leptin Signalling in Breast Cancer Cells

**DOI:** 10.1371/journal.pone.0158218

**Published:** 2016-07-14

**Authors:** Antonio Pérez-Pérez, Flora Sánchez-Jiménez, Teresa Vilariño-García, Luis de la Cruz, Juan A. Virizuela, Víctor Sánchez-Margalet

**Affiliations:** 1 Department of Medical Biochemistry and Molecular Biology and Immunology, UGC Clinical Biochemistry, Virgen Macarena University Hospital, University of Seville, Seville, Spain; 2 UGC Clinical Oncology, Virgen Macarena University Hospital, Seville, Spain; University of South Alabama, UNITED STATES

## Abstract

Obesity is a well-known risk factor for breast cancer development in postmenopausal women. High insulin and leptin levels seem to have a role modulating the growth of these tumours. Sam68 is an RNA-binding protein with signalling functions that has been found to be overexpressed in breast cancer. Moreover, Sam68 may be recruited to insulin and leptin signalling pathways, mediating its effects on survival, growth and proliferation in different cellular types. We aimed to study the expression of Sam68 and its phosphorylation level upon insulin and leptin stimulation, and the role of Sam68 in the proliferative effect and signalling pathways that are activated by insulin or leptin in human breast adenocarcinoma cells. In the human breast adenocarcinoma cell lines MCF7, MDA-MB-231 and BT-474, Sam68 protein quantity and gene expression were increased upon leptin or insulin stimulation, as it was checked by qPCR and immunoblot. Moreover, both insulin and leptin stimulation promoted an increase in Sam68 tyrosine phosphorylation and negatively regulated its RNA binding capacity. siRNA was used to downregulate Sam68 expression, which resulted in lower proliferative effects of both insulin and leptin, as well as a lower activation of MAPK and PI3K pathways promoted by both hormones. These effects may be partly explained by the decrease in IRS-1 expression by down-regulation of Sam68. These results suggest the participation of Sam68 in both leptin and insulin receptor signaling in human breast cancer cells, mediating the trophic effects of these hormones in proliferation and cellular growth.

## Introduction

Sam68, also known as KHDRBS1 (KH domain-containing, RNA-binding, signal-transduction-associated 1) is a member of the signal transduction activator of RNA (STAR) family of RNA-binding proteins (RBPs). As other members of this family, Sam68 contains a GRP33/Sam68/GLD1 (GSG or STAR) domain for the RNA binding activity [1,2], and can interact with both RNA targets and other proteins. According to the role of Sam68 as an RNA binding protein, it has been described that this protein modulates several steps of RNA metabolism [3], such as nuclear export and cytoplasmic utilization or translation of viral and cellular mRNAs [4,5] and regulation of alternative splicing, where Sam68 plays a key role [6].

In addition, this protein has been described as a scaffold protein recruited in various signal transduction pathways [7,8] linking signalling pathways and RNA metabolism regulation. Sam68, which was originally identified as the first specific target of the Src tyrosine kinase in mitosis [9,10], binds several proteins containing Src homology 3 (SH3) and Src homology 2 (SH2) domains through proline-rich sequences and tyrosine-phosphorylated residues, respectively. Sam68 splicing activity, RNA binding ability and localization are regulated by phosphorylation and other posttranslational modifications [11–15].

Sam68 has been previously implicated in cell proliferation, growth and differentiation processes through different mechanisms. In this sense, some studies have shown a role of Sam68 as a necessary factor for cellular cycle progression [16,17]. Moreover, alternative splicing of several proliferation-related genes as bcl-x(L), CD44, SGCE, centrophilin, and cyclin D1 [12,18–20] have been demonstrated to be regulated by Sam68. Thus, this protein has been shown to be related to cancer development and progression [21]. Most specifically, it has been shown that Sam68 is up-regulated in prostate cancer, where its down-regulation seems to promote the inhibition of cell proliferation and sensitization of cells to apoptosis induced by chemotherapeutic agents [22]. Moreover, Sam68 haploinsufficiency delays mammary tumor onset and multiplicity as it was shown in PyMT transgenic mice [23]. Silencing of Sam68 in the breast cancer cell lines have also shown inhibition of cell proliferation and anchorage-independent growth, up-regulation of cyclin-dependent kinase inhibitors, increased FOXO transcriptional activity and deactivation of the PI3K/Akt pathway [24].

The tyrosine phosphorylation of Sam68 has also been involved in cancer development or progression. Thus, Sam68 Tyr-phosphorylation has been reported in certain cancers where tyrosine kinases are induced. Indeed, it has been shown that Sam68 is a downstream substrate of the epithelial growth factor EGF through BRK phosphorylation [25] and its phosphorylation is also elevated in breast tumours tissues and cell lines [14]. Sam68 phosphorylation has been reported in other systems when they are stimulated by mitogenic and trophic hormones such as insulin and leptin, where it has been linked to cellular growth and proliferation through its participation in the main pathways activated by these hormones. Thus, Sam68 has also been related to the insulin-dependent MAPK and PI3K pathways activation, where it has been shown to be associated to Grb2, GAP and PI3K regulatory subunit, exerting a role in these pathways under insulin and also leptin stimulation in different cellular systems [26–28]. More recent studies have shown that Sam68 down-regulation avoided the complete leptin-dependent activation of MAPK and PI3K pathways in choriocarcinoma JEG3 cells, also preventing leptin-dependent cellular growth and proliferation [29].

Insulin and leptin are suggested links between obesity and breast cancer risk, as they may contribute to the malignant transformation of breast epithelial cells and cancer progression [30,31]. In this sense, they promote growth and proliferation in breast cancer cell lines [32–34]. Moreover, both hyperinsulinemia and hyperleptinemia are common findings in obesity-related breast cancer [35,36]. In this line, overexpression of insulin and leptin receptor have been found in postmenopausal breast cancer [37–39] and may influence prognosis and treatment response [40,41].

According to the previously described participation of Sam68 in insulin and leptin signalling [8], we aim to study the role of this protein in the signal transduction pathways that are activated by leptin and insulin to mediate their proliferative effect in breast cancer cells.

## Materials and Methods

### 2.1. Cell culture and treatments

MCF7, MDA-MB-231 and BT-474 cell lines were originally from American Tissue Type Culture (ATTC) and they were obtained from Dr. A. Lopez-Rivas (CABIMER, Seville, Spain) [42]. Since cell lines were originally obtained before 2010, cell lines were authenticated using STR analysis by the Instituto de Investigaciones Biomédicas (IIBM) “Alberto Sols” CSIC-UAM. MCF7 cells from passage-10 to -15 and MDA-MB-231 and BT-474 cells from passage -10 to -12 were grown in DMEM medium (Invitrogen) supplemented with 10% fetal calf serum (FCS), 100 U/mL penicillin, 100 g/mL streptomycin at 37°C in 5% CO_2_. Cells were treated with different concentrations of leptin and insulin (0–10 nM) during 10 min or 16h. The recombinant human leptin was provided by Sigma (Sigma Chemical); insulin Actrapid was purchased from Novo Nordisk. 1 nM dose of both hormones was used for the experiments, corresponding to the optimal dose-response in physiological concentrations [43,44].

The cell lisates were washed with cold PBS and solubilized for 30 min at 4°C in lysis buffer containing 20 mM Tris, pH 8, 1% Nonidet P-40, 137 mM NaCl, 1mM MgCl_2_, 1mM CaCl_2_, 10% glycerol, 1mM phenylmethylsulfonyl fluoride, and 0.4 mM sodium orthovanadate. Total protein levels were determined by the bicinchoninic acid method [45] using bovine serum albumin as standard.

### 2.2. Western blotting analysis

Lysates of Sam68 siRNA transfected cells were washed three times with lysis buffer. We added 40 uL of SDS-stop buffer containing 100mM of DTT to the immunoprecipitates followed by boiling for 5 min. The soluble supernatants were then resolved by 7–15% SDS-PAGE and electrophoretically transferred onto nitrocellulose membranes. The membranes were blocked with buffered saline–0.05% Tween 20 (PBST) containing 3% albumin for 1 h at 23°C. The blots were then incubated with primary antibody for 1 h (anti-β-tubulin 1:5000 and anti-Sam68 (C-20) 1:2000 from Santa Cruz Biotechnology; the anti phosphotyrosine (4G10) 1:1000 from Millipore; anti-phosphoMEK1/2 (pS217-pS221) and anti-phosphoERK1/2 (pT202-Y204/pT185-Y187) 1:1500 from Sigma Aldrich; anti-phosphoAKT (pS473) 1.3000 from BD Biosciences, Pharmigen; anti-P-RPS6KB1 (p70S6K) (pT389) 1:2000 was from Cell Signalling Technology; anti total insulin receptor substrate-1 (antiIRS-1) 1:2000 was from Santa Cruz. After the incubation with primary antibody, the membranes were washed in PBST, and further incubated with the corresponding secondary antibodies using horse radish peroxidase-linked anti-rabbit/anti-mouse 1:10000 immunoglobulin (GE healthcare). Bound horseradish peroxidase was visualized by a highly sensitive chemiluminescence system (SuperSignal from Pierce).

### 2.3. Transfection experiments

MCF7 cells were plated at a density of 2.5×10^5^ cells/mL onto six-well dishes containing 2 mL of DMEM plus 10% FCS. Cells were incubated for 24 h. Medium was replaced, and transfection of cells was performed using siRNA of Sam68 (Integrated DNA technology, Inc). Duplex Sequences: Forward, 5’-CGCAGAACAAAGUUACGAAGGCUAC-3’; reverse, 5’-GUAGCCUUCGUAACUUUGUUCUGCGUA-3’. Typically, 40 pmol of the Sam68 siRNA duplexes or the universal negative control duplex NC1 were transfected using 4 uL of LipofectAMINE (Life Technologies). The medium was replaced after 24 h using DMEM without FCS for 24 h more. Transfection analyses were performed by duplicate in each of at least three independent experiments.

### 2.4. Cell proliferation and viability assay

A commercial cell proliferation kit based on MTT colorimetric assay (Roche) was used according to the manufacturer’s instructions. After 16 hours of leptin and insulin stimulation in the Sam68 siRNA transfected cells, MTT labelling reagent was added to each well (final concentration 0.5 mg/mL). The cells were incubated for 4 hours and formazan dye was produced only in metabolic active cells. Then, the solubilisation solution was applied to the samples overnight and the absorbance was measured at 600 nM and compared to the negative control measurement.

### 2.5. RNA extraction and quantitative real-time-PCR (qRT-PCR) assay

Relative abundance of Sam68 and IRS-1 mRNA was determined by qRT-PCR. Total RNA was extracted from the three cell lines cultures using TRISURE reagent (Chomczynski. 1993). Concentration and purity of the isolated RNA were estimated spectrophotometrically at 260 and 280 nm. For cDNA synthesis, 5ug of total RNA was reverse transcribed at 55°C during 1 h using the Transcriptor first Strand cDNA synthesis Kit (Roche). qRT-PCR was performed using the following primers based on the sequences of the National Center for Biotechnology Information GenBank database: cyclophilin: forward, 5’-CTTCCCCGATACTTCA-3’; reverse, 5’-TCTTGGTGCTACCTC-3’; Sam68: forward, 5’-TTTGTGGGGAAGATTCTTGG-3’; reverse, 5’-GGGGGTCCAAAGACTTCAAT-3’. IRS-1: forward, 5’-ACCATGGGGACAAGCCCGGCG-3’; reverse, 5’-GGGGCTGCTGGTGTTGGAATC-3’. Quantitative RT-PCR Master Mix Reagent kit was obtained from Roche (Fast Start universal SYBR Green), and PCRs were performed on a Chromo 4 DNA Engine (Bio-Rad). A typical reaction contained 10 uM of forward and reverse primer, 3ul of cDNA, and the final reaction volume was 20 uL. The reaction was initiated by preheating at 50°C for 2 min, followed by heating at 95°C for 10 min. Subsequently, 41 amplification cycles were carried out as follows: denaturation 15 s at 95°C and 1 min annealing and extension at 58°C. The threshold cycle (CT) from each well was determined by the Opticon Monitor 3 Program. Relative quantification was calculated using the 2-ΔΔct method. For the treated samples, evaluation of 2-ΔΔct indicates the fold change in gene expression, normalized to a housekeeping gene (cyclophilin), and relative to the untreated control.

### 2.6. Data analysis

Experiments were repeated separately at least three times to assure reproducible results. Results are expressed as means ± standard deviation (SD) in arbitrary units (A.U.). Arbitrary units were calculated as normalized band intensity in Western blot analysis. Statistical analysis was performed using the Graph Pad Prism computer program (GraphPad Software). Statistical significance was assessed by ANOVA followed by different post hoc tests, as indicated in each figure. A p value <0.05 was considered statistically significant.

## Results

### Insulin and leptin up-regulate Sam68 mRNA expression and protein quantity in MCF7, MDA-MB-231 and BT-474 adenocarcinoma cells

Activation of signal receptors positively modulates the expression of different proteins that participate in signal transduction. Thus, we have previously shown an increase of Sam68 expression in response to insulin in CHO-IR cells and adipocytes [46] as well as in choriocarcinoma JEG3 cells in response to leptin [47].

**Fig 1:**
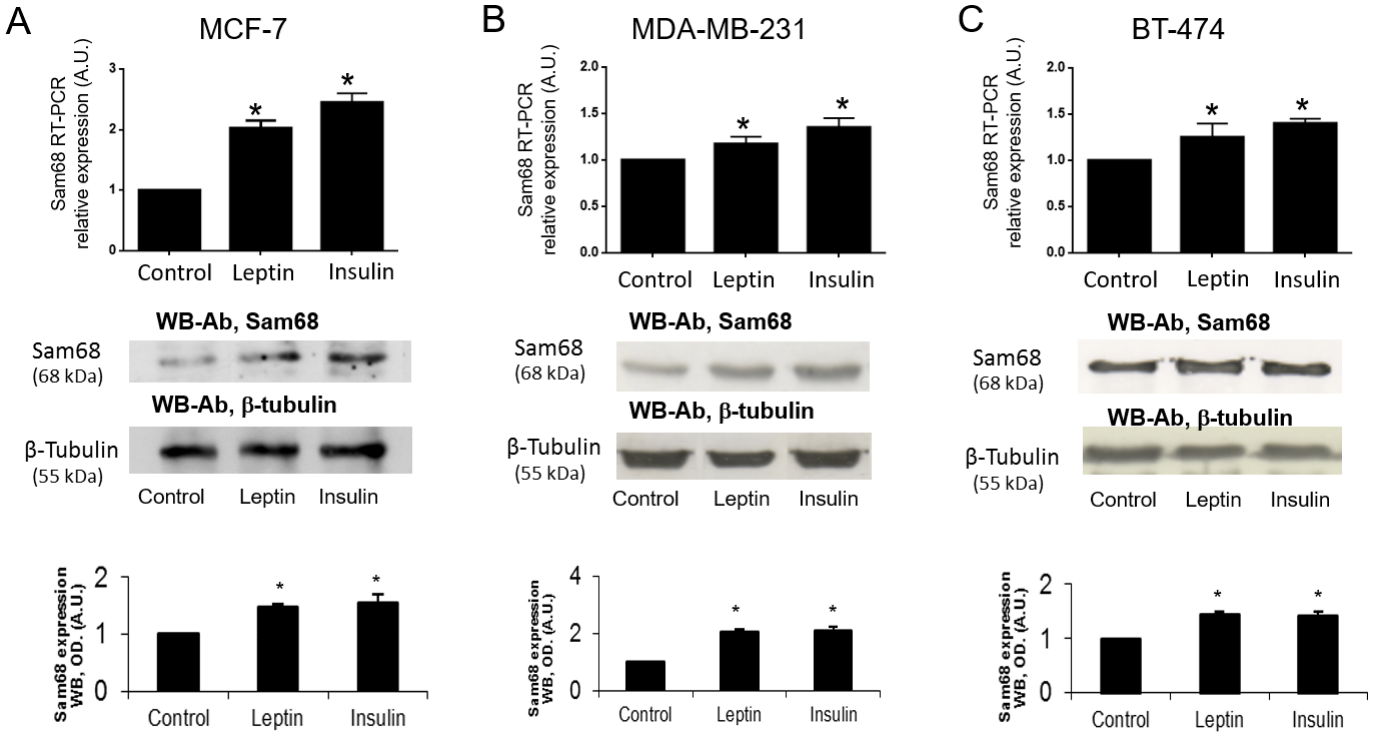
Leptin and insulin treatment for 24 h increases Sam68 expression measured as Sam68 mRNA quantification and Sam68 protein abundance in breast cancer cell lines. Cells were treated with 1 nM leptin dose or1 nM insulin dose during 24 h. Total RNA was extracted and Sam68 mRNA was quantified with qRT-PCR in independent experiments. Cyclophilin was used as internal standard. Results shown are from a representative experiment and are expressed as means ± SD for three independent experiments *p<0.05 versus control. Cells were lysed and proteins were separated on SDS-PAGE gels. Sam68 protein amount was determined by Western blot analysis. Total amount of protein in each sample was controlled by immunoblotting the same membranes with anti-β-tubulin antibodies. Fig 1a, b and c) RT-PCR relative expression results and western blot expression analysis for three different breast cancer cells lines (MCF7, MDA-MB-231 and BT-474 respectively). Bands densitometries are shown in a representative experiment of three independent ones for each cell line. Densitograms with SD are shown. *p< 0.05 versus control.

In order to check the effect of leptin and insulin on Sam68 expression in the three adenocarcinoma cell lines, they were independently incubated in the absence of serum with and without leptin or insulin (1 nM) for 24 h. We evaluated mRNA expression by means of qRT-PCR, using cyclophilin as an internal control for reaction efficiency. Similar experiments were performed to assess the effect of leptin and insulin on Sam68 protein amount by using Western blot analysis and antibodies that specifically recognize Sam68. The amount of total protein in every sample was controlled using anti-β-tubulin antibodies. As it is shown in Fig 1a, leptin and insulin stimulation for 24 h increased Sam68 expression about twice as much as the basal levels in MCF-7 cells. As shown in Fig 1b and 1c, both insulin and leptin enhanced the expression of Sam68 in MDA-MB-231 and BT-474 cells, increasing approximately 25% that from control with statistical significance for both insulin and leptin stimulus versus control.

### Insulin and leptin treatments stimulate Sam68 tyrosine phosphorylation in adenocarcinoma breast cells

We have previously implicated Sam68 as a signalling molecule in the LEPR system in human monocytes and lymphocytes [48,49] as well as in choriocarcinoma JEG3 cells [47]. Moreover, Sam68 has been considered as an insulin receptor substrate in CHO cells and in isolated rat adipocytes, where it mediates the insulin action [26,46,50,51]. Tyr-phosphorylation of Sam68 has been demonstrated in response to insulin and leptin [26,48].

**Fig 2:**
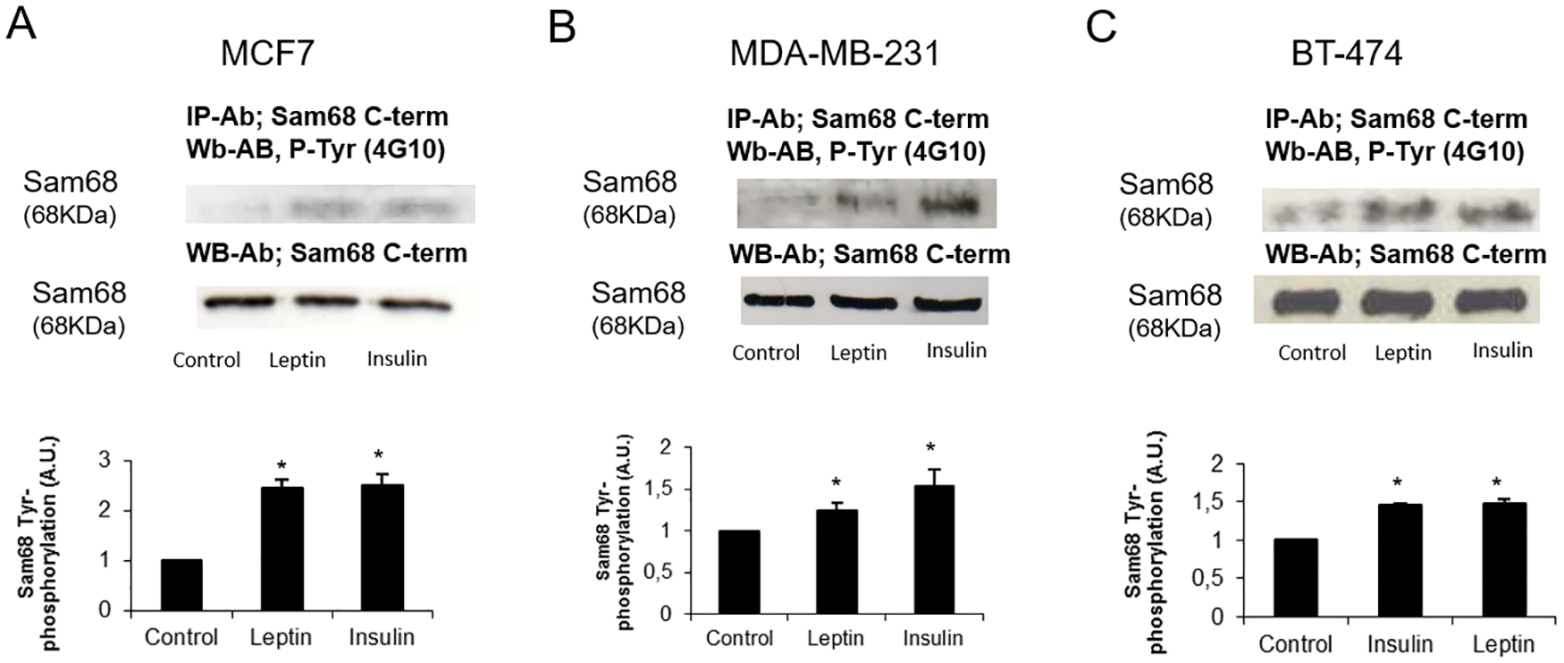
Leptin and insulin treatments of three different adenocarcinoma cellular lines increase Sam68 phosphorylation. Cells were incubated in the presence of leptin 1 nM or insulin 1 nM for 10 min, lysed and the soluble clarified cell lysates were subjected to immunoprecipitation with anti-Sam68 antibodies. Immunoprecipitates were resolved by SDS-PAGE and Western blot with anti-phosphotyrosine antibodies. The lysates were analyzed by immunoblot using the anti-Sam68 antibodies to control the amount of protein in every lane. Fig 2a, 2b and 2c) Immunoprecipitation results for, respectively, MCF7, MDA-MB-231 and BT-474 breast cancer cells.

We have now tested this possible implication of Sam68 in leptin and insulin signalling in adenocarcinoma cells by studying Tyr-phosphorylation mediated by these hormones. Cells were stimulated with a physiological 1 nM dose of leptin or insulin, for 10 min. Total extracts from control and treated MCF7, MDA-MB-231 and BT-474 cells were immunoprecipitated with C-terminal anti-Sam68 antibodies and analysed by means of immunoblotting with anti-phosphotyrosine antibodies. As shown in Fig 2a, 2b and 2c, Sam68 was tyrosine phosphorylated when the cells were incubated with both hormones, showing similar phosphorylating effect in MDA-MB-231 and BT-474 cells, and even higher than two fold increase in MCF7 cells. The amount of protein immunoprecipitated in every lane was controlled by immunoblotting with the same immunoprecipitating antibody.

### Inhibition of Sam68 expression by Sam68 siRNA prevents leptin and insulin stimulation of cellular growth in adenocarcinoma MCF7 cells

Biological effects of leptin and insulin in promoting growth and proliferation have been extensively reported in many different cell systems, including breast cancer cells [33,34]. Moreover, the functional role of Sam68 participating in leptin-dependent cellular growth and proliferation has previously been suggested in trophoblastic choriocarcinoma cells [29].

**Fig 3:**
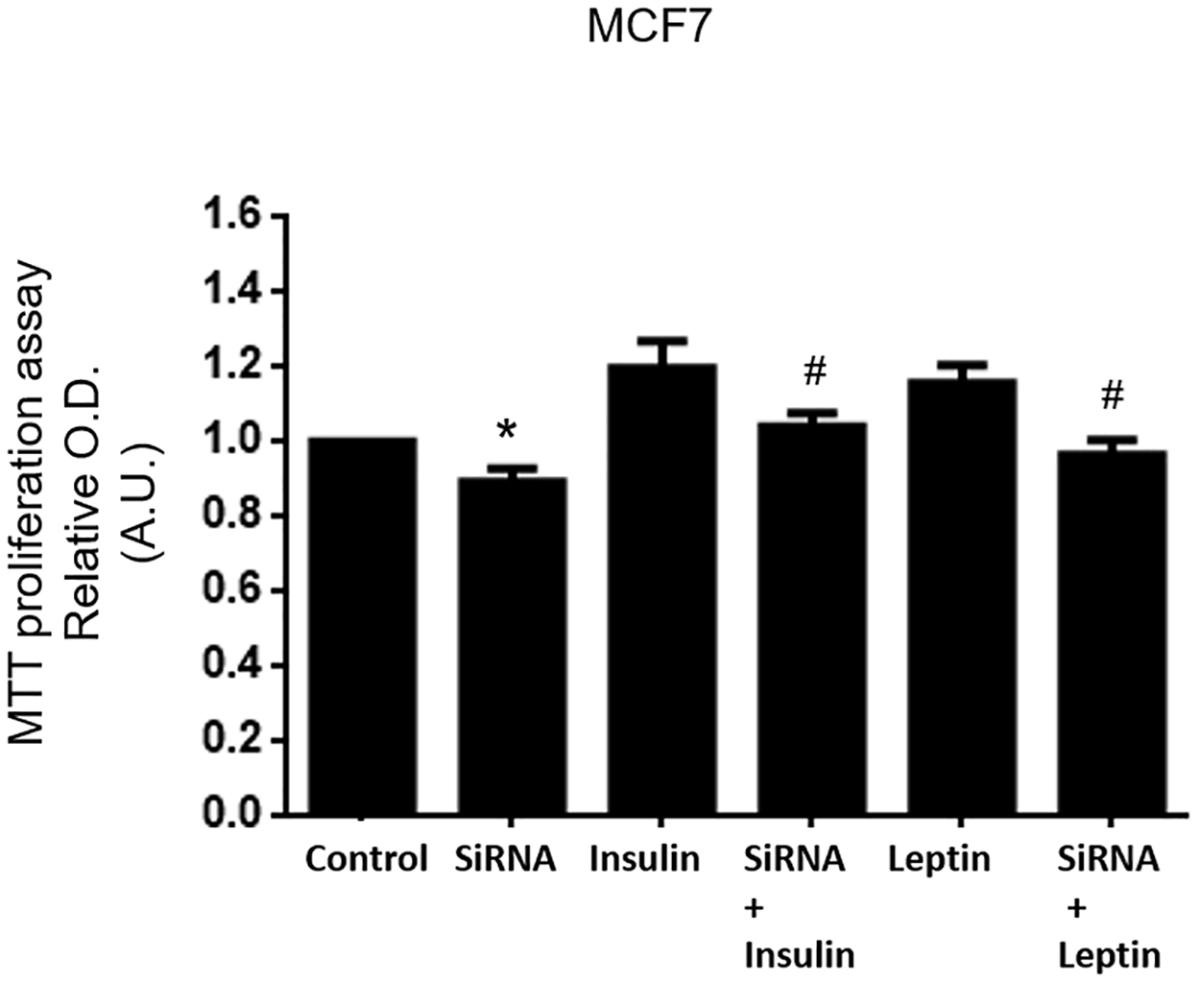
Leptin and insulin effects on cellular proliferation is impaired in Sam68 down-regulated adenocarcinoma MCF7 cells. MCF7 cells were transfected with Sam68 or NC1-scrambled negative control siRNA duplexes during 48 h. Cells were cultured for another 16 h in the presence or absence of 1 nM leptin or insulin. After that, the MTT reagent was added as indicated in Materials and Methods section. Data are expressed as means ± SD from four independent experiments, *P < 0.05 versus “Control”, #P <0.05 versus the corresponding non stimulated pair. “Control”: negative control siRNA transfected cells; “Sam68 siRNA”: Sam68 siRNA transfected cells without stimulus, “I”: negative duplex siRNA transfected and insulin stimulated cells; “Sam68 siRNA + I”: Sam68 siRNA transfected and insulin-stimulated cells; “L”: negative control siRNA transfected, leptin stimulated cells; “siRNA + L”: Sam68 siRNA transfected and leptin-stimulated cells.

To test the cellular effects of Sam68 down-regulation on the leptin and insulin trophic effects, we used MCF7 as the most responsive to hormone treatment cells. Besides, MCF7 cells have been successfully used as a pathophysiological approach for insulin and leptin-related breast cancer in previous works [33,34]. Cells were incubated in the absence or presence of biological dose of both hormones (1 nM) for 16 h after 48 h treatments with Sam68 siRNA or NC-duplexes siRNA as control. As shown in Fig 3, when Sam68 expression was decreased with siRNA, the effects of leptin and insulin activating the cellular metabolism were also impaired. The effect of Sam68 siRNA alone without hormonal stimulus also resulted in a decreased cell survival and proliferation as compared to control.

We have also confirmed these results in the other two breast cancer cell lines, where we have found very similar results. We are including the effect of Sam68 downregulation on both leptin- and insulin-stimulated cell proliferation in MDA-MB-231 and BT-474 cells in S1 Fig. Lowering the expression of Sam68 prevents the proliferative effect of both insulin and leptin.

### Sam68 down-regulation prevents leptin and insulin activation of signaling pathways that mediates their growth effects in adenocarcinoma MCF7 cells

**Fig 4:**
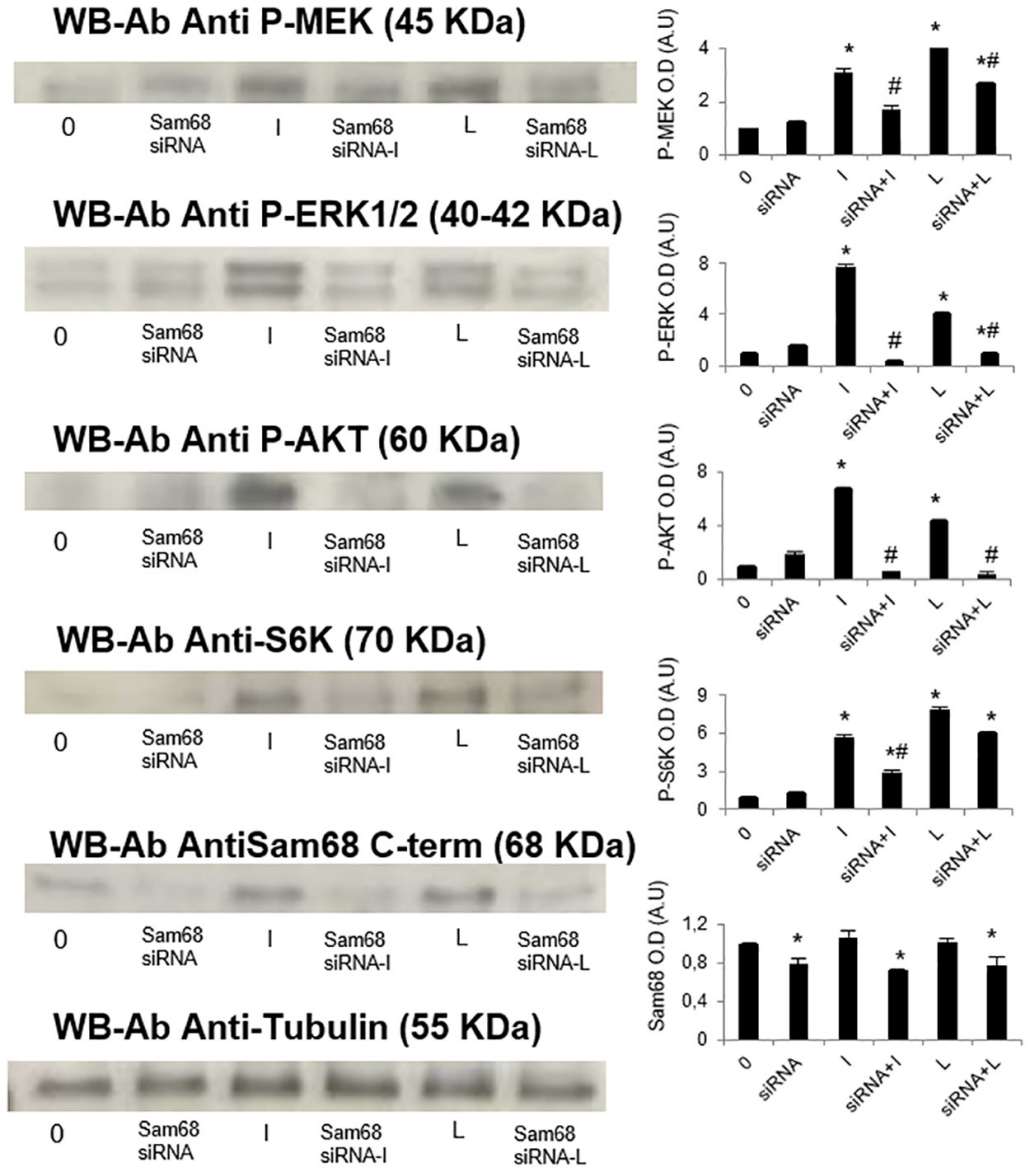
Sam68 down-regulation by Sam68 siRNA prevents the leptin and insulin-dependent activation of PI3K and MAPK pathways in MCF7 cells. MCF7 cells were transfected with Sam68 or NC1-scrambled negative control siRNA duplexes, during 24 h prior to stimulation with 1nM insulin or leptin for 10 min. Cells were lysed and soluble clarified cell lysates were separated by SDS–PAGE. A western blot analysis was performed by using anti-P-MEK1-2 and anti-P-MAPK1-2 antibodies to study leptin and insulin activation of the MAPK signaling pathway. Anti-P-AKT and anti-P-P70S6K antibodies were also used to study the leptin or insulin-dependent activation of the PI3K pathway. A western blot analysis was performed by using anti-Sam68 antibodies to control Sam68 down-regulation. Sample protein loading was controlled by using anti-β-tubulin antibodies. We show the corresponding densitometric analysis of three independent experiments as means ± SD, * p< 0.05 versus control “0”, # p< 0.05 versus leptin or insulin stimulated; “0”, negative duplex siRNA transfected, non-stimulated cells; “siRNA”, Sam68 siRNA transfected non-stimulated cells; “I”, negative duplex siRNA transfection and insulin-stimulated cells; “siRNA+I”, Sam68 siRNA transfected insulin-stimulated cells; “L”, negative duplex siRNA transfected leptin-stimulated cells; “siRNA+L”, Sam68 siRNA transfected leptin-stimulated cells.

Leptin and insulin are well known promoting factors of cellular growth and proliferation through phosphatidylinositol-3 kinase (PI3K) and the mitogen-activated protein kinase (MAPK) signalling pathways in different biological systems. To check the effect of Sam68 down-regulation on insulin and leptin-dependent phosphorylation of the main proteins of these signalling pathways, we used adenocarcinoma MCF7 cells as it was shown to have better response to insulin and leptin. Cells were firstly transfected using Sam68 siRNA or NC1 negative control siRNA duplexes and incubated in the absence or presence of insulin/leptin during 10 minutes as previously indicated in Material and Methods. Both anti-Sam68 and anti-β-tubulin antibodies were used as control of Sam68 down-regulation and loading control respectively. We measured the activation of MAPK pathway by employing antibodies that specifically recognize the phosphorylated forms of the kinases MEK1-2 and ERK1-2. As shown in Fig 4, the insulin and leptin-mediated MEK1-2 Ser phosphorylation was significantly reduced in cells where Sam68 was down-regulated. A similar effect was observed in the leptin and insulin activation of ERK1–2 proteins by Thr/Tyr phosphorylation, which was almost completely abolished by decreasing the expression of Sam68.

We also measured the activation of the central kinase of PI3K pathway, i.e. AKT, studying the effects of Sam68 down-regulation on the insulin and leptin mediated AKT phosphorylation. In addition, leptin and also insulin,stimulate P70S6K phosphorylation downstream to AKT activation, to promote the stimulation of protein synthesis. As shown in Fig 4, both P70S6K and AKT phosphorylation by insulin and leptin were impaired in cells that were treated with Sam68 siRNA to down-regulate Sam68 expression.

Similar results were observed in the other breast cancer cell lines employed in the present study. The insulin and leptin activation of both AKT and ERK signalling pathways are prevented by downregulation of Sam68 expression in MDA-MB-231 and BT-474 (S2 Fig).

**Fig 5:**
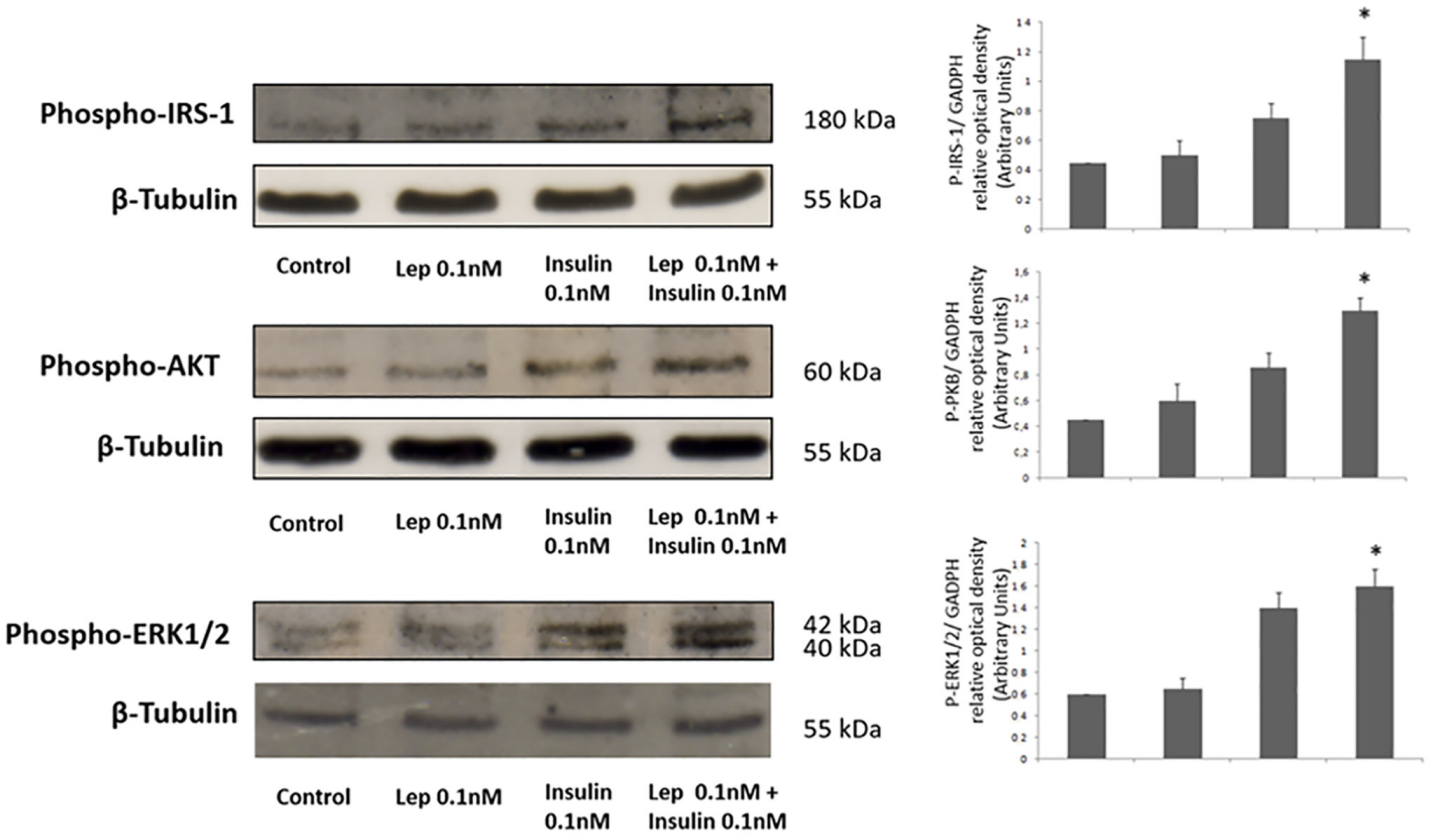
Leptin and insulin act synergistically activating PI3K and MAPK pathways in MCF7 cells. MCF7 cells were incubate in the presence or absence of suboptimal concentrations of insulin or leptin (0.1 nM) alone or simultaneously for 10 min. Cells were lysed and soluble clarified cell lysates were separated by SDS–PAGE. A western blot analysis was performed by using anti-P-IRS-1, anti-P-AKT and anti-P-ERK1-2 antibodies to study leptin and insulin activation of signaling pathways. A western blot analysis was performed by using anti-β-tubulin antibodies to control sample protein loading. We show the corresponding densitometric analysis of three independent experiments as means ± SD, * p< 0.05 versus leptin or insulin alone.

Since both insulin and leptin activate the same signalling pathways, we wanted to study whether both hormones might have a synergistic effect on the same signalling pathway. As shown in Fig 5, suboptimal concentrations (0.1 nM) of both leptin and insulin produced little phosphorylation signal in AKT and ERK signalling pathways. However, when both hormones were present at 1 nM concentration at the same time a greater effect was observed, suggesting that insulin and leptin act synergistically in MCF7 cells.

### Sam68 down-regulation results in a decrease of mRNA and protein abundance of the Insulin Receptor Substrate-1 in breast cancer cells

**Fig 6:**
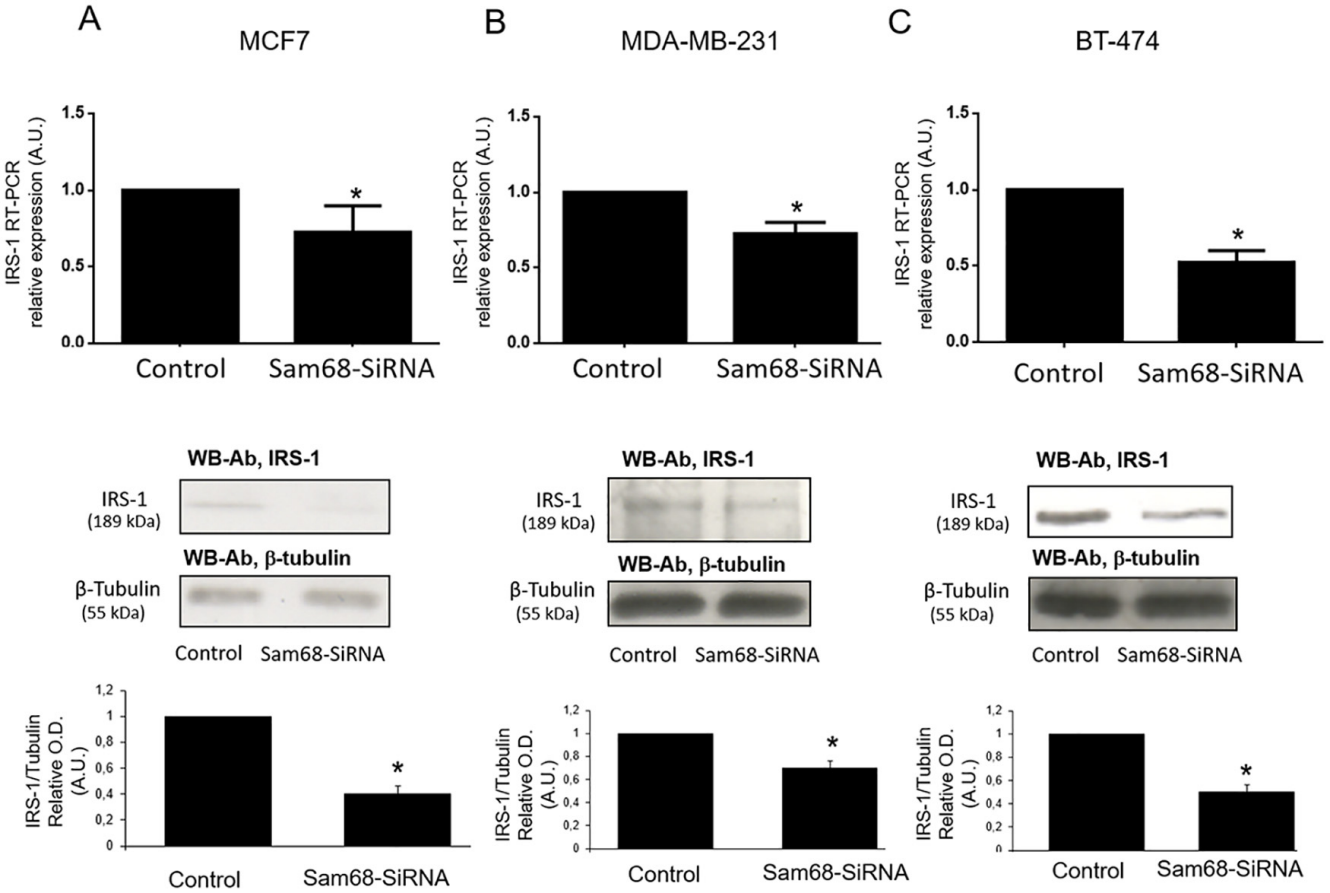
IRS-1 expression and protein abundance is reduced in Sam68 siRNA transfected breast cancer cells. Cells were transfected using NC-scrambled siRNA duplexes as negative control or Sam68 siRNA during 24 horas. Cells were lysed and the soluble clarified cell lysates were subjected to total RNA extraction. IRS-1 mRNA was quantified with qRT-PCR using cyclophilin as internal standard. The soluble clarified cell lysates were immunoblotted with anti-IRS-1 antibodies, using anti-β-tubulin antibodies as loading control. Results shown are from a representative experiment and are expressed as means ± SD for three independent experiments *p<0.05 versus control. A. MCF-7 cells; B, MDA-MB-231; C, BT-474 cells.

To connect Sam68 expression with the mechanistic effect that exerts over the main pathways that mediates growth and proliferation under leptin and insulin stimulation, we next focus on the effect of siRNA Sam68 down-regulation on IRS-1 basal expression in the three breast cancer cell lines: MCF7, MDA-MB-231 and BT-474 cells. As it was demonstrated using qRT-PCR as well as immunoblotting analysis with anti-IRS-1 antibodies (Fig 6), down-regulation of Sam68 in the three cell lines, significantly decreased the expression of IRS-1, as compared to negative control siRNA transfected cells.

## Discussion

A function of Sam68 in cancer has previously been suggested [21]. More precisely, Sam68 has been involved in breast cancer tumorigenesis [52] and cell proliferation, where Sam68 upregulation in a large cohort of high proliferating breast tumours has also been found [24]. Since we have found that both insulin and leptin increase Sam68 expression in breast cancer cell lines, the hyperinsulinemia and hyperleptinemia [35,36] that occur in obese women may contribute to the overexpression of Sam68 found in the tumours from patients with obesity-related breast cancer.

Sam68 has been shown to have a dual role as an RNA-binding protein and as a docking protein in different systems [8], participating in signal transduction of different receptors, such as insulin [26], leptin [27], T-cell receptor [53], as well as EGF [25], HGF/Met [54] or TNFR1 [55]. In addition, Sam68 has also been involved in posttranscriptional gene regulation [3], where some posttranscriptional modifications of Sam68 such us Tyr-phosphorylation [9], Ser/Thr phosphorylation [12], acetylation [14], methylation [13] or SUMOylation [15] may modify Sam68 ability to participate in some cellular events. In the present work we have confirmed the Tyr-phosphorylation of Sam68 upon insulin and leptin stimulation in three different breast cancer cell lines. Moreover, the insulin and leptin-mediated increase in Sam68 expression further supports the possible role of Sam68 in the signalling of both hormones.

Moreover, it has previously been found that tyrosine phosphorylation of Sam68 inhibits its ability to bind RNA in different systems [10,27,47,56]. In this sense, signal transduction has been found to be linked to RNA metabolism in different physiological and pathophysiological scenarios, including breast cancer [25]. Since we have found that tyrosine phosphorylation of Sam68 by insulin or leptin stimulation decreases its binding capacity to RNA also in adenocarcinoma cells (data not shown), some of the effects of these hormones in breast cancer cells may be mediated, at least in part, by modulation of RNA metabolism.

The proliferative effects of elevated insulin levels have been demonstrated in many different cellular system, being involved in cancer development and progression [57], where it has been proposed to promote proliferation in the human breast cancer line MCF-7 by facilitating the transit of cells through G1[43]. In addition, leptin has been considered a cytokine associated to breast cancer proliferation too [58], with an important role in the breast cancer cellular microenvironment [59,60]. We have confirmed the growth promoting effect of insulin and leptin in breast cancer cells, as well as the importance of Sam68 expression to exert this effect, since down-regulation of Sam68 impairs the proliferative action of both hormones.

As previously shown in many different cellular systems, also in tumoral cells activation of MAPK and PI3K signalling pathways are well known to promote proliferation, cell survival and cellular growth [61,62]. Leptin and insulin have previously been demonstrated to activate these pathways in cancer and, specifically, breast cancer cells [41,63]. Now, by using siRNA strategy, we have demonstrated not only that Sam68 is mediating both insulin and leptin stimulated cellular metabolism, but also its participation in the leptin and insulin dependent activation of MAPK and PI3K signalling pathways in breast cancer cells. Moreover, the effect of insulin and leptin activating these signalling pathways seems to be synergic since simultaneous stimulation with suboptimal concentrations (0.1 nM) produced a greater activation, similar to that observed when maximal concentration of leptin or insulin alone were employed.

Regarding the mechanism whereby Sam68 may mediate the activation of these pathways, it has previously been reported that Sam68 is associated with the SH3 domains of Grb2 in a constitutive way and may interact with the SH2 domains of GAP after insulin stimulation in HTC-IR cells [51] suggesting a role of Sam68 in the MAPK pathway. Moreover, the association of Tyr-phosphorylated Sam68 with the regulatory subunit of PI3K have been previously reported in hepatocytes and adipocytes in response to insulin [26] and in peripheral blood mononuclear cells in response to leptin [27]. This interaction may enhance the activation of PI3K pathway, which may support a role of Sam68 also in the activation of this pathway by these hormones. More importantly, IRS-1 is a key protein linking insulin and leptin stimulated PI3K and MAPK signaling pathways [64]. Sam68 has been previously demonstrated to interact with IRS-1 [65] according to the previously suggested role as adaptor protein in signal transduction in both leptin and insulin systems [8]. Since Sam68 seems to regulate IRS-1 expression, as we observed in the Sam68 down-regulation experiments, the role of Sam68 stimulating PI3K and MAPK signaling pathways may be mediated by IRS-1. As a consequence, the inhibition of cell growth, either basal or insulin/leptin stimulated, may also be mediated by the inhibition of IRS-1 expression, since the down-regulation of IRS-1 is known to decrease cell growth rate [66]. Since Sam68 downregulation led to a decrease in both mRNA and protein content of IRS-1, the effect of Sam68 on IRS-1 expression should be at the transcription level. However, the mechanism whereby Sam68 may modulate IRS-1 expression is intriguing, and remains to be investigated.

Sam68 has previously been considered as a multifunctional player in human cancer, and more specifically in breast cancer [21,52]. The phosphorylation of Sam68 by Brk in Met receptor signalling is one of the known molecular mechanisms of breast cancer progression [54]. Since insulin and leptin increase the expression of Sam68, this protein may be a point of convergence for different signals involved in breast cancer growth and progression. In fact, increased Sam68 expression has already been found in breast tumours. Now, we have shown that Sam68 plays a role in leptin and insulin signal transduction pathways in three different adenocarcinoma breast cancer cell lines. Moreover, we are suggesting new mechanisms that link Sam68 expression and phosphorylation with obesity-associated breast cancer. Thus, Sam68 may be mediating the proliferative effects of insulin and leptin, by activation of MAPK and PI3K signalling pathways, at least in part modulating the expression of IRS-1.

In conclusion, Sam68 seems to participate in both leptin and insulin receptor signalling in human breast cancer cells, mediating the trophic effects of these hormones in proliferation and cellular growth.

## Supporting Information

S1 Fig Leptin and insulin effects on cellular proliferation is impaired in Sam68 down-regulated MDA-MB-231 and BT-474 cells. MDA-MB-231 cells (A) or BT-474 cells (B) were transfected with Sam68 or NC1-scrambled negative control siRNA duplexes during 48 h. Cells were cultured for another 16 h in the presence or absence of 1 nM leptin or insulin. After that, the MTT reagent was added as indicated in Materials and Methods section. Data are expressed as means ± SD from four independent experiments, *P < 0.05 versus “Control”, #P <0.05 versus the corresponding non stimulated pair. “Control”: negative control siRNA transfected cells; “Sam68 siRNA”: Sam68 siRNA transfected cells without stimulus, “I”: negative duplex siRNA transfected and insulin stimulated cells; “Sam68 siRNA + I”: Sam68 siRNA transfected and insulin-stimulated cells; “L”: negative control siRNA transfected, leptin stimulated cells; “siRNA + L”: Sam68 siRNA transfected and leptin-stimulated cells.
(TIF)

S2 Fig Sam68 down-regulation by Sam68 siRNA prevents the leptin and insulin-dependent activation of PI3K and MAPK pathways in MDA-MB-231 and BT-474 cells. MDA-MB-231 cells (A) or BT-474 cells (B) were transfected with Sam68 or NC1-scrambled negative control siRNA duplexes, during 24 h prior to stimulation with 1nM insulin or leptin for 10 min. Cells were lysed and soluble clarified cell lysates were separated by SDS–PAGE. A western blot analysis was performed by using anti-P-AKT, anti-ERK1-2 antibodies to study leptin and insulin activation of these signaling pathways. Sample protein loading was controlled by using anti-β-tubulin antibodies. We show the corresponding densitometric analysis of three independent experiments as means ± SD, * p< 0.05 versus control “0”, # p< 0.05 versus leptin or insulin stimulated. “0”, negative duplex siRNA transfected, non-stimulated cells; “siRNA”, Sam68 siRNA transfected non-stimulated cells; “I”, negative duplex siRNA transfection and insulin-stimulated cells; “siRNA+I”, Sam68 siRNA transfected insulin-stimulated cells; “L”, negative duplex siRNA transfected leptin-stimulated cells; “siRNA+L”, Sam68 siRNA transfected leptin-stimulated cells.
(TIF)
