# Supplementary figures and images for: Sam68 Mediates the Activation of Insulin and Leptin Signalling in Breast Cancer Cells
Source: PLoS One. 2016 Jul 14;11(7):e0158218. doi: 10.1371/journal.pone.0158218 (PMC4944952; doi:10.1371/journal.pone.0158218)

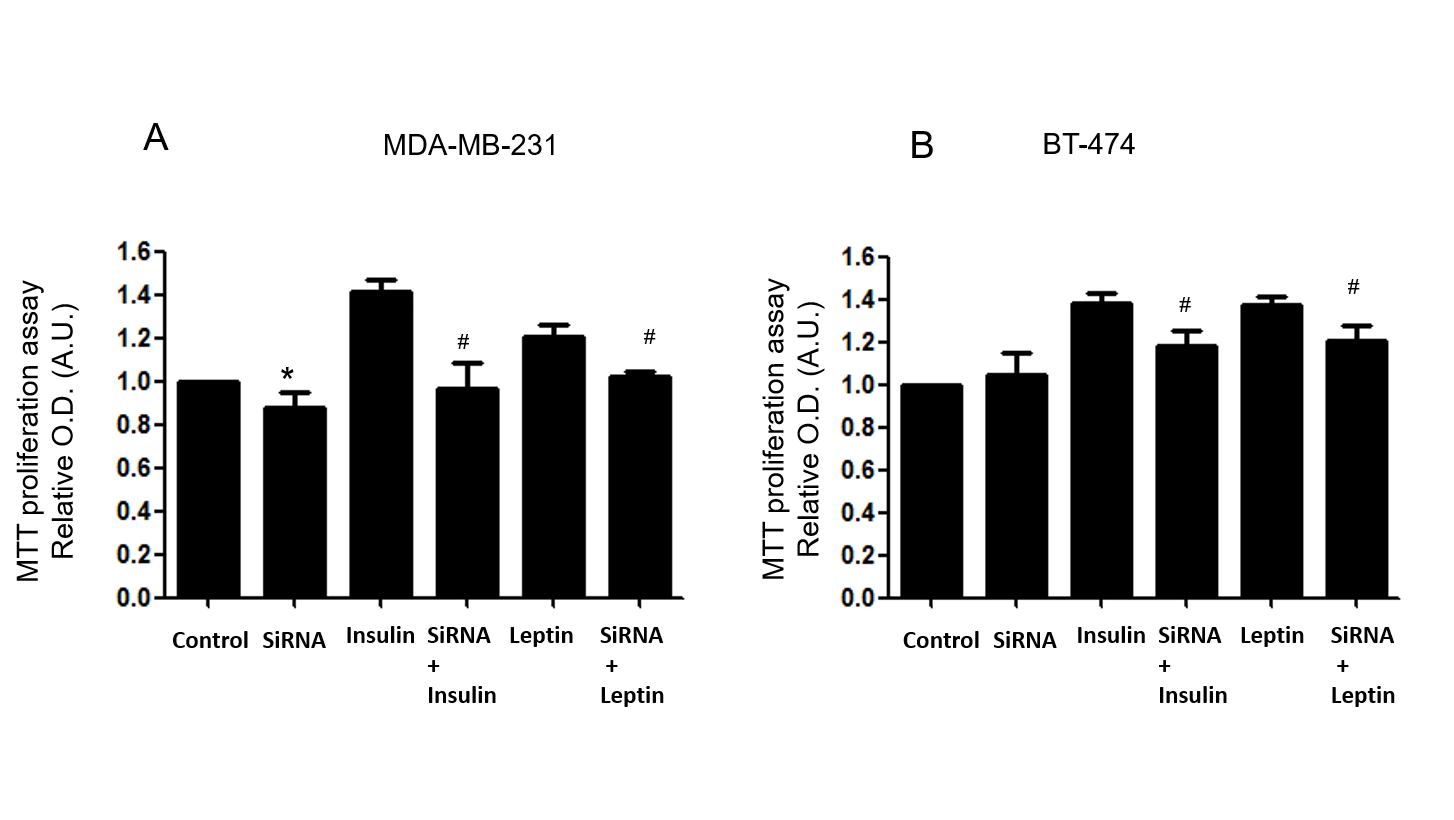

Supplement: S1 Fig — MDA-MB-231 cells (A) or BT-474 cells (B) were transfected with Sam68 or NC1-scrambled negative control siRNA duplexes during 48 h. Cells were cultured for another 16 h in the presence or absence of 1 nM leptin or insulin. After that, the MTT reagent was added as indicated in Materials and Methods section. Data are expressed as means ± SD from four independent experiments, *P < 0.05 versus “Control”, #P <0.05 versus the corresponding non stimulated pair. “Control”: negative control siRNA transfected cells; “Sam68 siRNA”: Sam68 siRNA transfected cells without stimulus, “I”: negative duplex siRNA transfected and insulin stimulated cells; “Sam68 siRNA + I”: Sam68 siRNA transfected and insulin-stimulated cells; “L”: negative control siRNA transfected, leptin stimulated cells; “siRNA + L”: Sam68 siRNA transfected and leptin-stimulated cells. (TIF) [file pone.0158218.s001.tif]

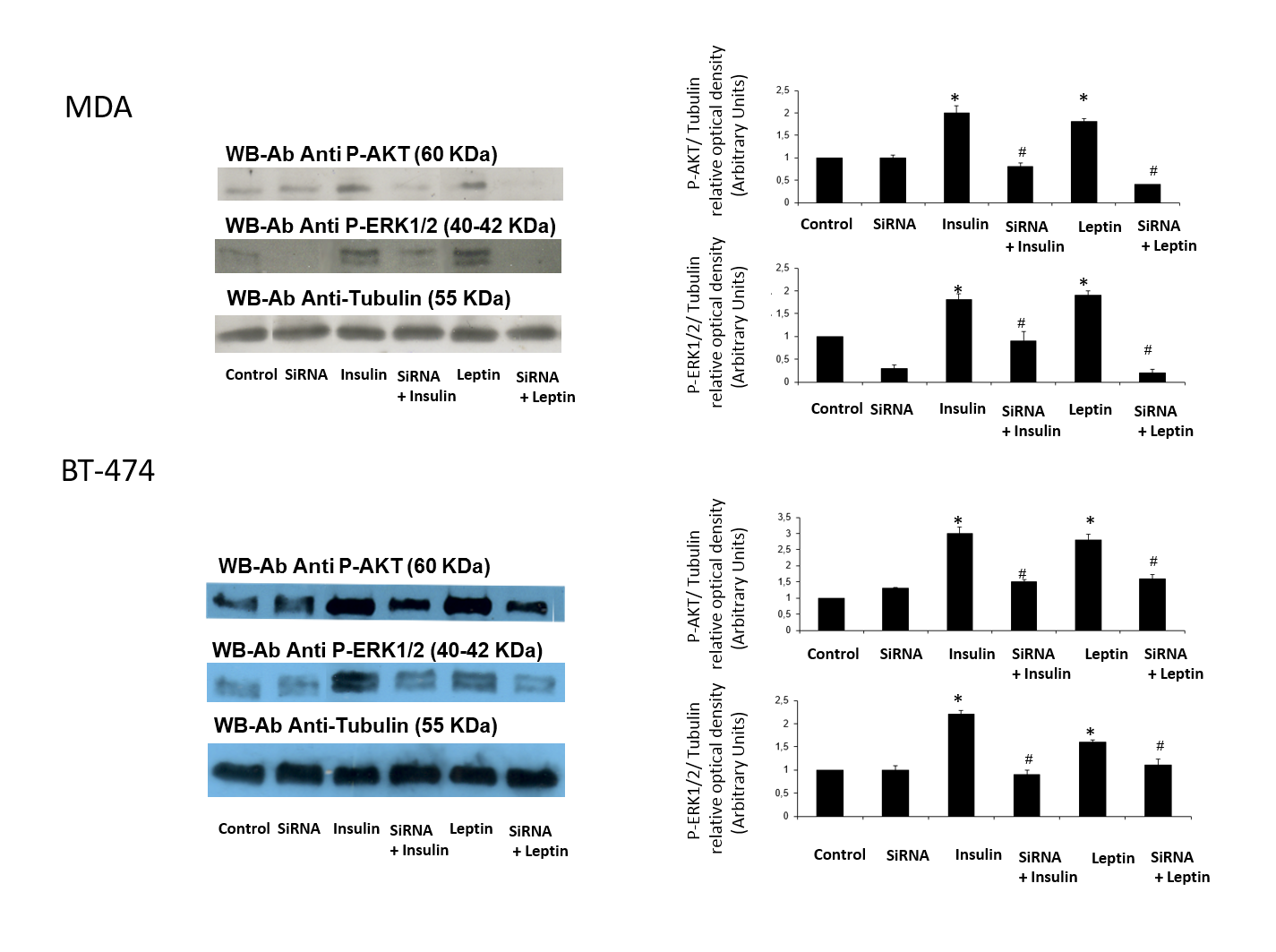

Supplement: S2 Fig — MDA-MB-231 cells (A) or BT-474 cells (B) were transfected with Sam68 or NC1-scrambled negative control siRNA duplexes, during 24 h prior to stimulation with 1nM insulin or leptin for 10 min. Cells were lysed and soluble clarified cell lysates were separated by SDS–PAGE. A western blot analysis was performed by using anti-P-AKT, anti-ERK1-2 antibodies to study leptin and insulin activation of these signaling pathways. Sample protein loading was controlled by using anti-β-tubulin antibodies. We show the corresponding densitometric analysis of three independent experiments as means ± SD, * p< 0.05 versus control “0”, # p< 0.05 versus leptin or insulin stimulated. “0”, negative duplex siRNA transfected, non-stimulated cells; “siRNA”, Sam68 siRNA transfected non-stimulated cells; “I”, negative duplex siRNA transfection and insulin-stimulated cells; “siRNA+I”, Sam68 siRNA transfected insulin-stimulated cells; “L”, negative duplex siRNA transfected leptin-stimulated cells; “siRNA+L”, Sam68 siRNA transfected leptin-stimulated cells. (TIF) [file pone.0158218.s002.tif]
